# Ecophysiological Traits of Invasive C_3_ Species *Calotropis procera* to Maintain High Photosynthetic Performance Under High VPD and Low Soil Water Balance in Semi-Arid and Seacoast Zones

**DOI:** 10.3389/fpls.2020.00717

**Published:** 2020-07-02

**Authors:** Rebeca Rivas, Vanessa Barros, Hiram Falcão, Gabriella Frosi, Emília Arruda, Mauro Santos

**Affiliations:** ^1^Laboratório de Fisiologia Vegetal, Departamento de Botânica, Universidade Federal de Pernambuco, Recife, Brazil; ^2^Laboratório de Anatomia Vegetal, Departamento de Botânica, Universidade Federal de Pernambuco, Recife, Brazil

**Keywords:** C_3_ photosynthesis, evergreen, leaf anatomy, oxidative stress, plant biomass, sugars metabolism

## Abstract

The evergreen C_3_ plant *Calotropis procera* is native to arid environments. Thus, it grows under high vapor pressure deficit (VPD), intense light, and severe drought conditions. We measured several ecophysiological traits in *C. procera* plants growing in semi-arid and seacoast environments to assess the attributes that support its photosynthetic performance under these contrasting conditions. Gas exchange analysis, primary metabolism content, nutrients, the antioxidant system, and leaf anatomy traits were measured under field conditions. In the semi-arid environment, *C. procera* was exposed to a prolonged drought season with a negative soil water balance during the 2 years of the study. *Calotropis procera* plants were exposed to a positive soil water balance only in the rainy season in the seacoast environment. The leaves of *C. procera* showed the same photosynthetic rate under high or low VPD, even in dry seasons with a negative soil water balance. Photosynthetic pigments, leaf sugar content, and the activity of antioxidant enzymes were increased in both places in the dry season. However, the anatomical adjustments were contrasting: while, in the semi-arid environment, mesophyll thickness increased in the driest year, in the seacoast environment, the cuticle thickness and trichome density were increased. The ability to maintain photosynthetic performance through the seasons would be supported by new leaves with different morpho-anatomical traits, with contrasting changes between semi-arid and seacoast environments. Furthermore, our results suggest that an efficient antioxidative system and leaf sugar dynamics can contribute to protecting the photosynthetic machinery even under severe drought.

## Introduction

Changes in global rainfall patterns have resulted in more frequent drought across the semi-arid environments of the world (IPCC Climate Change, 2018). Several species, mainly C_3_ plants, can be severely damaged and coming under threat of extinction under this scenario when considering the higher frequency of prolonged dry seasons and the low efficiency of water use among these species (Oliveira et al., 2014; Santos et al., 2014). High ecophysiological plasticity is an important trait under climatic changes. Thus, species with high performance in water use efficiency and under intense light and vapor pressure deficit (VPD) could have an advantage over others (Ribeiro et al., 2009; Lawson et al., 2014), and also, changes in their leaf sugar metabolism and enzymatic antioxidant system could enable them to avoid ROS damage (Noctor et al., 2014). Moreover, it has been showing that morpho-anatomical features can effectively minimize the deleterious effects on the photosynthetic machinery (Tezara et al., 2011; Frosi et al., 2013; Santos et al., 2014).

Several plant species present outstanding performance and overcome limited resources, such as low water availability in semi-arid and arid regions, even during prolonged drought (Oliveira et al., 2014). In this context, some species have the ability to colonize environments with different available resources (Tezara et al., 2011; Frosi et al., 2013). Many exotic species introduced into ecosystems not only survive but adapt themselves and compete with native species (Kimberling, 2004). Invasiveness can be explained by several functional traits (Funk and Vitousek, 2007; Funk et al., 2016), such as high relative growth rate (Penuelas et al., 2010), increased efficiency in uptake and use of resources, and phenotypic plasticity (Funk and Vitousek, 2007; Davidson et al., 2011).

In South America, *Calotropis procera*, an evergreen C_3_ species from Asia and African arid areas, has become invasive by colonizing environments with different characteristics after having been used for ornamental and forage purposes (Tezara et al., 2011; Frosi et al., 2013; Rivas et al., 2017). The invasive ability of *C. procera* is supported by its high drought tolerance (Tezara et al., 2011; Frosi et al., 2013; Rivas et al., 2017) and salinity tolerance (Khan et al., 2007). This species could be an important source of data for improving new crop varieties to increase the efficiency of water use, an important feature in the face of climate change. The ecophysiological performance of *C. procera* under different environments in South America is known (Tezara et al., 2011; Frosi et al., 2013); gas exchange analyses and even some morpho-anatomical aspects have been measured. On the other hand, it is still not clear which traits are determinants for this performance over most C_3_ plants under the same environmental conditions. In the present study, our setup is different, with a robust approach that gathers data on several traits at the same time in two contrasting local environments, one drier and other with higher VPD. This work aims to answer four key questions. Firstly, does *C. procera* invest in the same morpho-anatomical traits in both environments? Second, does leaf biochemistry change differently over the seasons in the two environments, and does this change lead to increased CO_2_ assimilation? Third, does the photosynthetic rate have the same intensity in both rainy and dry seasons? Forth, what are the traits that determine this photosynthetic metabolism performance?

Thus, this study investigated what physiological and anatomical attributes allowed *C. procera* to keep its photosynthetic activity high even under high VPD and low soil water availability. This study was conducted in two environments, semi-arid and seacoast, analyzing the performance of gas exchange, leaf sugar metabolism, the antioxidative system, and leaf anatomy attributes for two consecutive years. We have two hypotheses: first, *C. procera* has higher CO_2_ assimilation in a seacoast environment than in a semi-arid environment, especially in the dry season, when water availability in the semi-arid region is extremely low compared to plants near the ocean, with a strong role played by the morpho-anatomic changes; second, the antioxidative system and leaf sugars change their activity and content in the leaves, respectively, in dry season to support the leaf photosynthetic machinery.

## Materials and Methods

### Plant Material

*Calotropis procera* (Aiton) W. T. Aiton (Apocinaceae) has a wide geographical distribution around the world. This species has the ability to disperse large numbers of seeds in the wind, grows rapidly, and has hermaphroditic flowers (Hassan et al., 2015). It is native from northwestern Africa (Senegal and Mauritania) to southwest Asia via the Arabian Peninsula, Afghanistan, Pakistan, and India (Hassan et al., 2015). It is commonly found in pastures and coastal zones and near dunes, highways, and urban land lots (Hassan et al., 2015).

### Study Sites

This study was carried out under natural conditions in two different environments – semi-arid and seacoast – in Pernambuco State, where *C. procera* populations were found. The semi-arid environment is located at Serra Talhada city (7°57′8.37″S, 38°17′54.07″W) in the Northeast of Brazil. The climate is BSh, according to Köppen’s classification (Alvares et al., 2013), with an average annual rainfall of approximately 750 mm, concentrated between December and May (Santos et al., 2014). The soil is classified as red-yellow podzolic soil, and its chemical composition is shown in Table 1. The seacoast environment is located at Maria Farinha beach (7°50′33.35″S, 34°50′20.63″W) in the Northeast of Brazil. The climate is As, according to Köppen’s classification (Alvares et al., 2013), with an average annual rainfall of approximately 2,050 mm, concentrated between April and July. The soil is classified as quartz sand, and its chemical composition is shown in Table 1.

**TABLE 1:** Soil analysis for semi-arid and seacoast regions in northeast Brazil.

**Region**	**Coarse sand**	**Fine sand**	**Silt**	**Clay**	**Water available**	**pH**	**P**	**K**	**Al**	**Ca**	**Mg**	**Cl**
	**(%)**						**(mg dm^–3^)**	**(cmol_c_ dm^–3^)**				**mg kg^–1^**
Semi-arid	43.00	32.00	13.00	12.00	4.52	6.6	>40	>0.45	0.00	5.05	0.95	87
Seacoast	57.00	32.00	7.00	4.00	4.65	7.4	19	0.04	0.00	1.95	0.90	192

The *Calotropis procera* individuals selected were of the same size and were evaluated in the dry and rainy seasons of 2012 and 2013 in both environments. In the semi-arid region, the rainy season was evaluated in April (2012) and March (2013), while the dry season was evaluated in November of both years. In the seacoast region, the rainy season was evaluated in June (2012) and May (2013), while the dry season was evaluated in November in both 2012 and 2013.

### Measurements

#### Leaf Osmotic Potential and Soil Water Balance

Osmotic potential (Ψ_s_) was measured using leaf tissues (500 mg), which were stored frozen at −20°C. The tissues were thawed and centrifuged at 10,000 *g* for 10 min at 4°C to extract the cell sap. The sap osmolarity (c) was measured using a vapor pressure osmometer (Silva et al., 2010). Ψ_s_ was calculated by the formula: Ψ_s_ = −c×2.58×10^−3^.

The soil water balance (SWB) was calculated according to Thornthwaite and Mather (1955) from air temperature and rainfall. We assumed an available soil water storage capacity of 75 and 100 mm for the semi-arid and seacoast regions, respectively.

This species of the Apocinaceae family produces a large amount of latex, which prevents the precise measurement of water potential by the Scholander method. In addition, due to the fact that the study was totally under field conditions, it was not possible to measure the water potential by the psychrometric method or to measure the relative water content.

#### Gas Exchange Daily Curves

Gas exchanges were measured using an infrared gas analyser (IRGA, ADC, model LCi-pro; Hoddesdon, United Kingdom) on the youngest fully expanded leaf throughout the day (totaling 20 measuring points). Water use efficiency (WUE) was calculated by dividing *A* by transpiration rate (*E*). For each measure, environment light radiation was used to adjust the photosynthetic photon flux density (PPFD). Vapor pressure deficit (VPD) was calculated for each measuring point throughout the day according to Campbell and Norman (1998).

#### Biochemistry, Nutrients, and Oxidative Stress

Mature, but not senescent, fully expanded leaves were collected at 1500 h in the rainy and dry seasons of both years (2012 and 2013) for each region (semi-arid and seacoast). All the leaves were immediately frozen in liquid nitrogen and stored in a freezer at −20°C. The leaves were used to determine the contents of Soluble sugar (SS), reducing sugar (RS), sucrose, fructose, free amino acids (FAA) total soluble protein (TSP), proline, chlorophyll a (Chl a), chlorophyll b (Chl b), carotenoids (Car), malondialdehyde (MDA), and hydrogen peroxide (H_2_O_2_). For soluble sugar, reducing sugar, sucrose, fructose, and free amino acids, a 50-mg fresh mass of leaves was used in ethanolic extraction. The soluble sugars were measured according to DuBois et al. (1956), using d (+) glucose as a standard and 490 nm absorbance. Reducing sugar (RS) was measured according to Nelson (1944) and Somogyi (1951). Sucrose was measured according to Handel (1968), using sucrose as standard and 660 nm absorbance. Fructose was measured according to Foreman et al. (1973), using fructose as standard and 410 nm absorbance. Free amino acids were analyzed according to Moore and Stein (1948), using a solution of 1 mM glycine, glutamic acid, phenylalanine, and arginine as standard and 570 nm absorbance. The insoluble fraction of the total soluble sugars extracted from leaves was used to determine the starch content. The pellet was hydrolyzed for 1 h with 10 units of amyloglucosidase, and the resulting sugars were analyzed (DuBois et al., 1956).

For the total soluble protein determination, a 170-mg fresh mass of leaves was macerated in 100 mM potassium phosphate buffer at pH 6.5 and measured according to Bradford (1976), using 0.1% bovine serum albumin (v/v) as standard and 595 nm absorbance. To determine proline, a 300-mg fresh mass of leaves was used and the proline content measured by the acidic ninhydrin method (Bates et al., 1973) using proline as standard and 520 nm absorbance. For chlorophyll a, chlorophyll b, and carotenoids, 150 mg of fresh leaf material was macerated in 2 mL of acetone (80%) with calcium carbonate (CaCO_3_) to prevent chlorophyllase activity. Samples were filtered and read at 470.0, 648.8, and 663.2 nm absorbance. Additionally, 710-nm non-specific absorbance was read to correct for color, turbidity, and contaminating compounds, since pigments are not read in this wavelength. Final pigment concentrations were calculated as described by Lichtenthaler and Buschmann (2001). To assess cellular damage, we measured the accumulation of malondialdehyde (MDA) and hydrogen peroxide (H_2_O_2_) using 100 mg of tissue. MDA content was assessed with the thiobarbituric acid (TBA) test, which measures MDA as a final product of lipid peroxidation. The amount of MDA-TBA complex (red pigment) was calculated using an extinction coefficient of 155 mM1 cm1 (Cakmak and Horst, 1991). Hydrogen peroxide (H_2_O_2_) accumulation was measured spectrophotometrically after reacting with KI (Alexieva et al., 2001).

To measure the leaf content of nitrogen (N), phosphorus (P), potassium (K^+^), sodium (Na^+^) and chlorine (Cl^–^), 200 mg of mature leaves were collected in both of the areas and in the different seasons of the year. The material was digested in sulfuric acid solution (H_2_SO_4_) in a digester block at 350°C to obtain the sample extract (Thomas et al., 1967). The total N content was determined by extract titration using HCl after adding boric acid and a colorimetric indicator (Thomas et al., 1967). The P content was determined spectrophotometrically (Spectrophotometer 600S, FEMTO, SãoPaulo, Brazil) according to Murphy and Riley (1962) using a P-concentration curve. The K^+^ content was determined by flame photometry (DM-62, Digimed, São Paulo, BR) using a 5-ppm K^+^ solution as standard. Chlorine (Cl-) was quantified according to Lacroix et al. (1970).

For oxidative stress, three antioxidant enzymes were analyzed - superoxide dismutase (SOD, E.C. 71.15.1.1), catalase (CAT, E.C. 1.11.1.6), and ascorbate peroxidase (APX, E.C.1.11.1.11) were analyzed following the methodologies proposed by Giannopolitis and Ries (1977), Havir and McHale (1987), and Nakano and Asada (1981), respectively. We used 350, 250, and 250 mg fresh weight leaf to determine the activities of SOD, CAT, and APX, respectively.

### Leaf Anatomy

#### Light Microscopy

From four plants, we collected two leaves per plant (the same criteria as for gas exchange) on each day of field visit. The leaves were fixed in formalin–acetic–alcohol (1:1:18, respectively) for 48 h and kept in ethanol 70% (Johansen, 1940). Subsequently, the leaves were dehydrated through a tertiary butanol series and encased in paraffin, and then 10-μm sections were obtained using a rotatory microtome (Zeiss, model Hyrax M55). All of the sections were subsequently stained with safranin-astra blue (Kraus et al., 1998). For epidermal dissociation, the samples were submitted to the methodology proposed by Strittmatter (1973) and then stained with 1% safranin. Photomicrographs were obtained using a Leica DM 500 light photomicroscope. For the quantitative evaluation of leaf samples, the image analysis software Image J Version 1.47r was used. From each plant, we analyzed 20 randomly selected microscopic fields of view for the transverse sections and paradermal sections (adaxial and abaxial epidermis). We measured the leaf thickness (LT), mesophyll thickness (MT), palisade parenchyma thickness (PPT), spongy parenchyma thickness (SPT), epidermal thickness (ET), and cuticle thickness (CT) on the transverse sections. In addition, we measured the stomatal density (SD; number of stomata/unit area), trichome density (TD), and epidermal cell density (ECD). The Stomatal index was obtained as SI = (SN/(NT + ECN)) × 100 and the trichome index as TI = (TN/(NT + ECN)) × 100 (Salisbury, 1928), in which SN is the number of stomata, ECN is the epidermal cell number, and TN is the number of trichomes.

#### Scanning Electron Microscopy

Fragments (0.5 cm^2^) were collected from the central region of fully expanded young leaf without senescence in the dry and rainy seasons in both years for the semi-arid and seacoast regions. The samples were fixed in glutaraldehyde (2.5%), 0.1 M cacodylate buffer (pH 7.2), and post-fixed with osmium tetroxide and 0.1 M cacodylate buffer (1:1). Subsequently, samples were dehydrated through acetone series (30–100%), subjected to a critical point (CPD 030 BAL-TEC), and metalized using gold (Leica-EM SCD500). The epidermis was examined in frontal view using a scanning electron microscope (QUANTA 200FEG).

### Statistical Analysis

Data were subjected to factorial ANOVA with season and year as independent factors. When necessary, means were compared using the Student-Newman-Keuls test at 5% significance. Data were analyzed using the software Statistica 8.0 (StatSoft. Inc., Tulsa, OK 74104, United States).

A principal component analysis (PCA) was performed to check for possible clusters and define the most important variables during the separation of groups. Data were transformed (ranging) for standardization due to different scale magnitudes. The substantial correlation values were determined for each attribute in relation to principal components (PC), and the level of importance of each variable was determined by eigenvector values (McGariga et al., 2000). The level of importance of each PC was determined by the broken-stick method, where eigenvalues exceeding the expected values were kept for interpretation. Analyses were performed using the software Fitopac 2.1.2.85 (Shepherd, 2010).

## Results

### Soil Water Balance and Environmental Conditions

**FIGURE 1:**
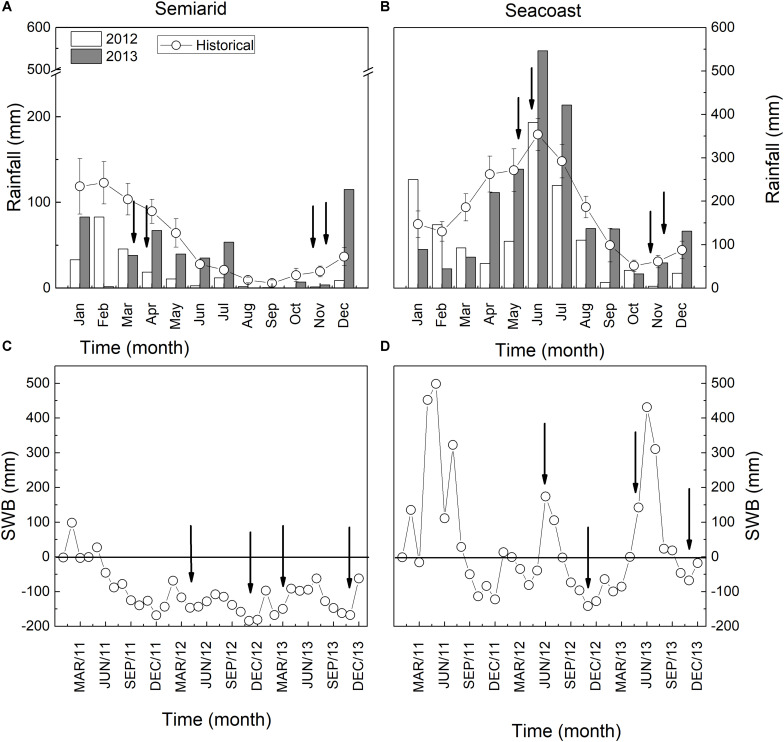
Rainfall and soil water balance (SWB) in semi-arid **(A,C)** and seacoast **(B,D)** environments.

The rainfall for the semi-arid and seacoast regions is expected to be 700 and 2,100 mm per year, respectively (Figures 1A,B). However, the semi-arid region accumulated 220 mm in 2012 and 446 mm in 2013, decreases of 69 and 36%, respectively, compared to historical values (Figure 1A). Consequently, SWB was negative in 2012 and 2013 (Figure 1C). In the seacoast region, 1,476 mm rainfall accumulated in 2012, 30% less than expected, while in 2013, the rainfall was compatible with expectations (2,166 mm) (Figure 1B). This scenario reflected positive SWB values in the rainy season and negative values in the dry season (Figure 1D).

**FIGURE 2:**
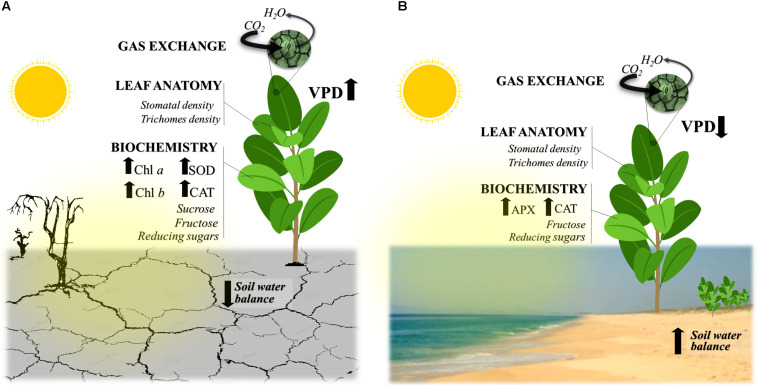
Major traits supporting the tolerance of *Calotropis procera* plants to the environmental conditions in the two studied environments. Although under contrasting environments, **(A)** semi-arid and **(B)** seacoast, the evergreen C_3_ *C. procera* plants maintained intense gas exchange over 2 years, independent of the water balance in the soil (SWB) and variation of the vapor pressure deficit (VPD). We highlight the changes over the studied period of the antioxidative system (SOD, superoxide dismutase; CAT, catalase; APX, ascorbate peroxidase), the modulation of photosynthetic pigments (chlorophyll *a*, *b*; Chl *a*, *b*), and the leaf morphoanatomical traits, such as stomatal and trichome density throughout the seasons, as well as the dynamics of sugars, as discussed in detail in the article.

**FIGURE 3:**
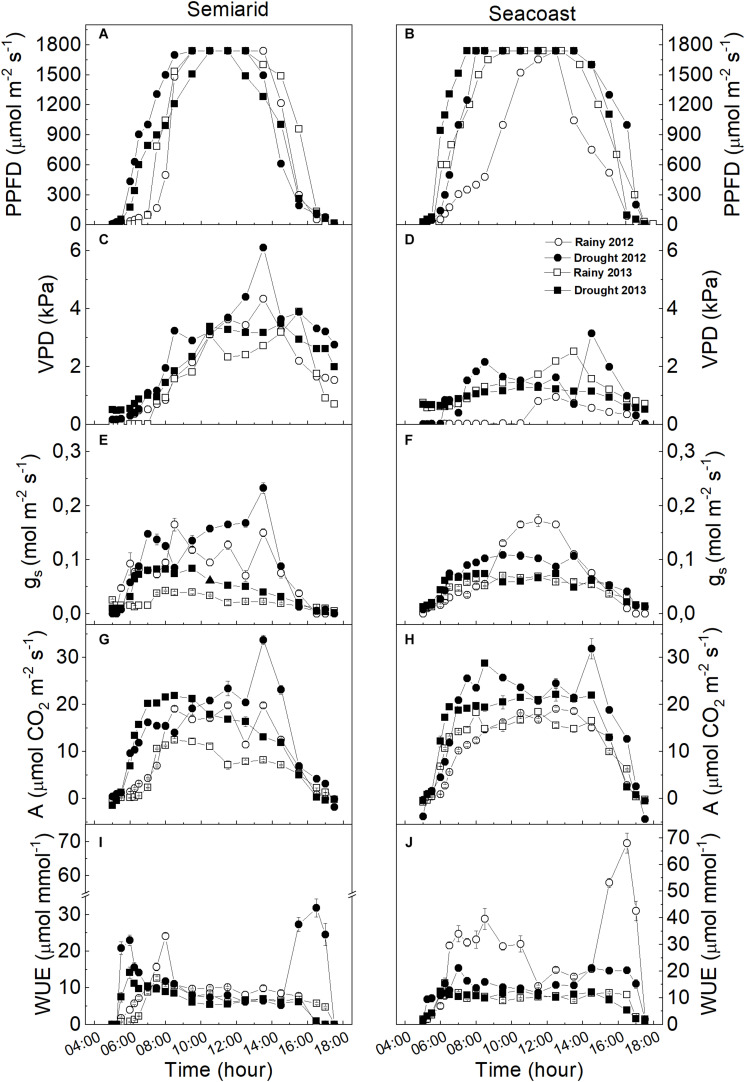
Daily gas exchange curve for *Calotropis procera* in semi-arid and seacoast environments in the rainy and drought seasons of 2012 and 2013. **(A,B)** Photosynthetic photon flux density (PPFD), **(C,D)** vapor pressure deficit (VPD), **(E,F)** stomatal conductance (g_s_), **(G,H)** net assimilation of CO_2_ (*A*), **(I,J)** water use efficiency (WUE) (*n* = 4 ± SE).

Our results showed that the plants of C. *procera* under both studied environments had intense dynamics throughout the seasons, adjusting morphoanatomical, biochemical, and physiological aspects of their leaves (Figure 2). During the diurnal period (sunrise – 0500 h and sunset – 1700 h local time), *C. procera* was exposed to VPD and PPFD changes (Figures 3A–D). In the early morning, PPFD increased until 0830 h, remained constant until 1400 h and then declined progressively until 1700 h (Figures 3A,B). In the ascending phase of the curve, the dry season showed higher PPFD compared to rainy seasons in both areas (Figures 3A,B). Concerning VPD, the peaks were observed during dry season 2012 in both the semi-arid and seacoast regions (Figures 3C,D).

### Leaf Osmotic Potential

In the semi-arid region, leaf osmotic potential decreased by 15% in dry season 2013 in relation to rainy season. No significant difference was observed during 2012 in the semi-arid region or the seacoast region (Table 2). In general, the values were lower in the semi-arid environment when compared with the seacoast, despite the high concentration of NaCl present in the leaf tissue.

**TABLE 2:** Osmotic potential (−Ψ_S_) in *Calotropis procera* during drought and rainy seasons (2012 and 2013) in semi-arid and seacoast regions (*n* = 4 ± SE).

**Location**	**Season**	**Year**	**−Ψ_S_ (MPa)**
Semiarid	Drought	2012	1.33 ± 0.03 AB
		2013	1.41 ± 0.02 B
	Rainy	2012	1.34 ± 0.04 AB
		2013	1.22 ± 0.03 A
Seacoast	Drought	2012	1.07 ± 0.03 NS
		2013	1.14 ± 0.03
	Rainy	2012	1.18 ± 0.02
		2013	1.20 ± 0.04

*Means followed by different letters are significantly different according to the Student-Newman-Keuls test (*P* < 0.05).*

### Gas Exchange Daily Curves

*Calotropis procera* showed similar behavior for gas exchange in both studied regions. In general, *g*_s_ and *A* increased progressively until 0830 h, following by increased PPFD and decreased until sunset (Figures 3A,B,E–H). The highest *A* values were observed in the dry season in both the semi-arid and seacoast regions in both years, with peaks around 1400 h in the dry season of 2012, at same time as VPD peaks (Figures 3C,D,G,H). Interestingly, *g*_s_ had the same pattern as *A* only in the semi-arid region, while at the seacoast, at some moments, *g*_s_ was higher in the rainy season in 2012 compared to the respective dry season values (Figures 3E,F). The WUE values in the semi-arid region remain the same in both years and seasons, with higher values in the dry season of 2012, around 1400 h to 1700 h (Figure 3I). At the seacoast, the highest WUE values were in the rainy season of 2012 (Figure 3J).

### Biochemistry, Oxidative Stress, and Nutrients

**FIGURE 4:**
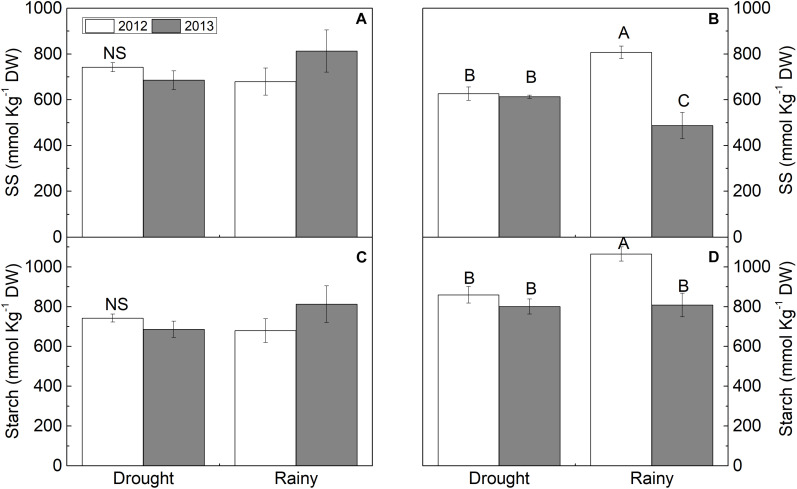
**(A,B)** soluble sugar (SS) and **(C,D)** starch in *Calotropis procera* under semiarid and seacoast environment in rainy and drought seasons of 2012 and 2013 (*n* = 4 ± S.E.). Values followed by different letters differ from each other by the Student Newman Keuls test (*P* < 0.05).

In spite of the prolonged drought in the semi-arid region, SS, starch (Figures 4A,I), and FAA (Supplementary Figure S1A) content were did not differ between the dry and rainy seasons in both years. However, plants in the dry season in both years had increases of 100 and 50% in fructose in 2012 and 2013, respectively, 134% (2012) and 100% (2013) in sucrose, and 300% in 2012 and 700% in 2013 in reducing sugars (RS) compared to the rainy season (*P* < 0.05) (Figures 4C,E,G). Inversely, TSP decreased (*P* < 0.05) in the dry season by 46% in 2012 and 73% in 2013 compared to the rainy season (Supplementary Figure S1E).

In the seacoast region, the solute contents differed between 2012 and 2013. In 2012, fructose, RS, and FAA increased by 28, 37, and 6% in the dry season, respectively (Figures 4D,H and Supplementary Figure S1B), while SS, starch, and TSP decreased by 22, 19, and 80%, respectively (Figures 4B,J and Supplementary Figure S1F). In the dry season of 2013, only SS content increased (26%), while fructose, sucrose, and TSP decreased by 36, 31, and 26%, respectively, compared to the rainy season (Figures 4B,D,F and Supplementary Figure S1F). The RS, starch, FAA, and proline content were maintained in both seasons of 2013 (Figures 4H,J and Supplementary Figures S1B,D).

**FIGURE 5:**
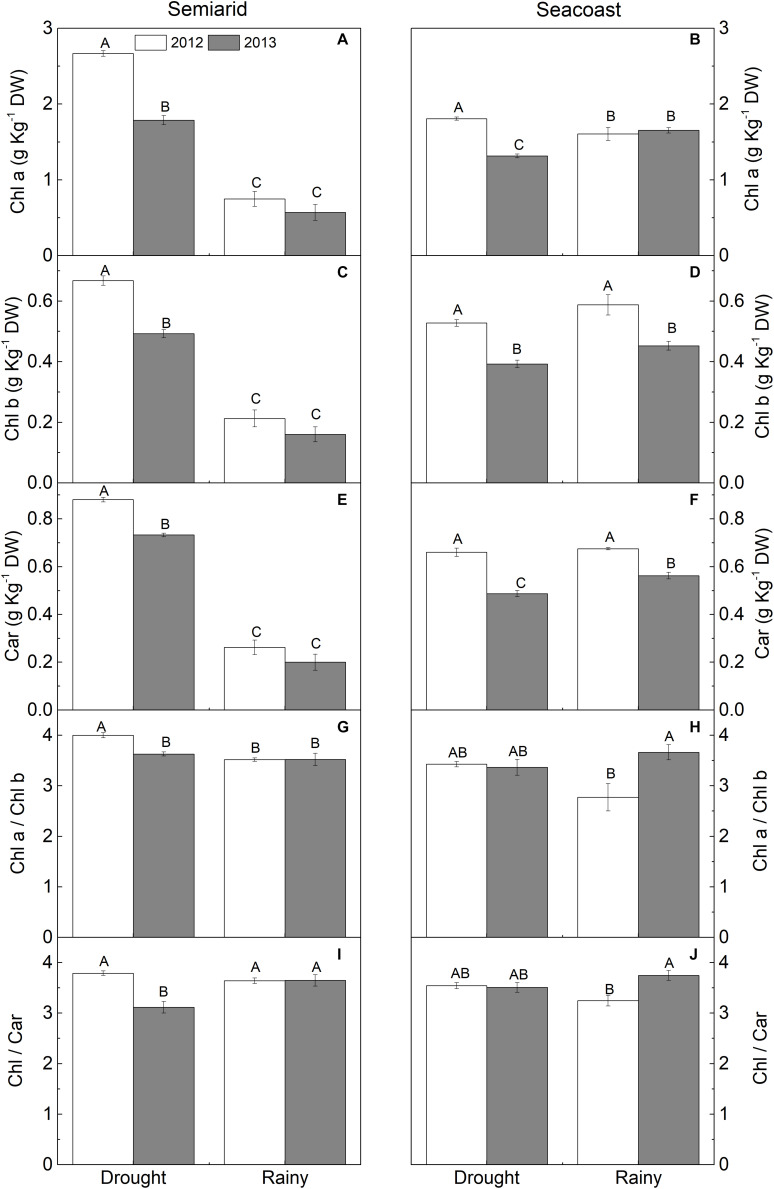
**(A,B)** Chlorophyll *a* (Chl *a*), **(C,D)** Chlorophyll *b* (Chl *b*), **(E,F)** carotenoids (Car), **(G,H)** Chl *a*/Chl *b*, and **(I,J)** Chl/Car in *Calotropis procera* under semi-arid and seacoast environments in rainy and drought seasons of 2012 and 2013 (*n* = 4 ± SE). Values followed by different letters differ from each other according to the Student-Newman-Keuls test (*P* < 0.05).

The photosynthetic pigment contents increased during the dry season in the semi-arid region to more than twice that in the rainy season in both years (Figures 5A,C,E). In addition, the Chl *a*/Chl *b* ratio increased by 14% in 2012 (Figure 5G). On the other hand, at the seacoast, Chl *a* increased by 12% in the dry season compared to the rainy season in 2012, while the inverse pattern was found in 2013, with decreases in Chl *a* (20%) and Car (14%) in the dry season compared to the rainy season (Figures 5B,F).

**FIGURE 6:**
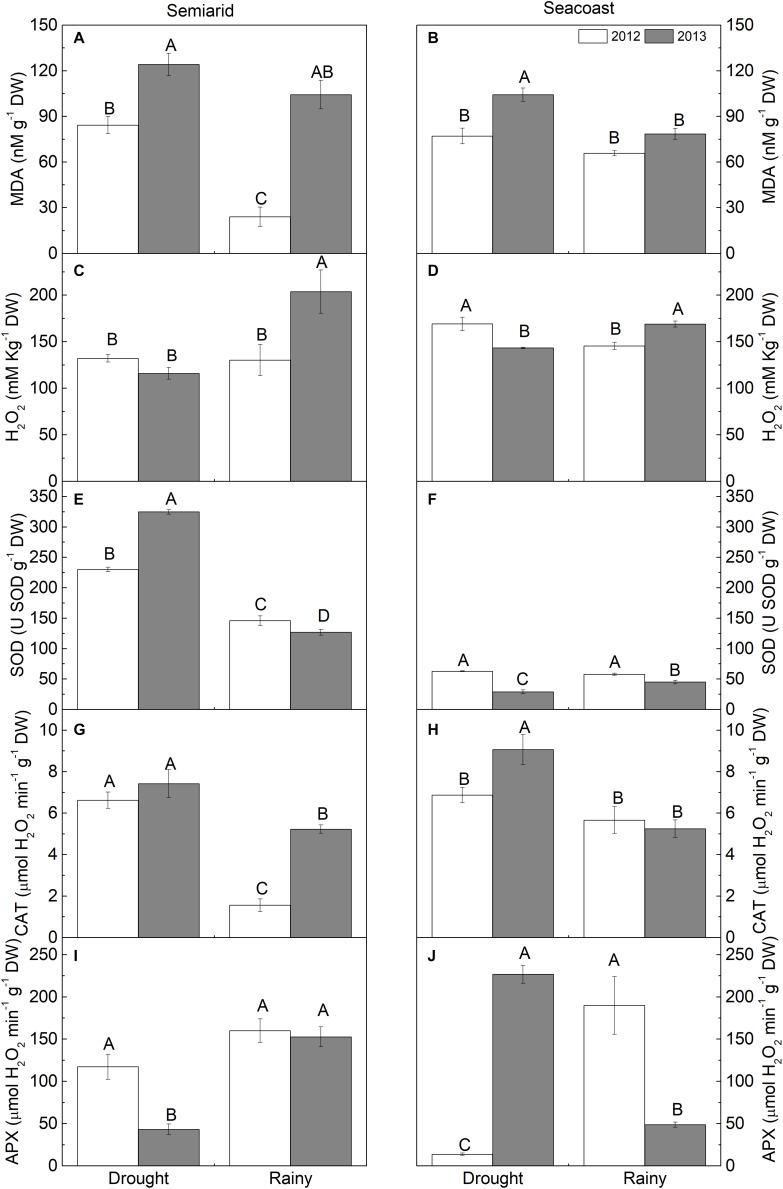
**(A,B)** Malondialdehyde (MDA), **(C,D)** hydrogen peroxide (H_2_O_2_), **(E,F)** superoxide dismutase (SOD), **(G,H)** catalase (CAT), and **(I,J)** ascorbate peroxidase (APX) in *Calotropis procera* under semi-arid and seacoast environments in rainy and drought seasons of 2012 and 2013 (*n* = 4 ± SE). Values followed by different letters differ from each other according to the Student-Newman-Keuls test (*P* < 0.05).

Regarding the oxidative stress, plants in the semi-arid region had increased MDA content during the dry season, 2.5 times in 2012, while H_2_O_2_ decreased by 43% in 2013 in the same season (Figures 6A,C). Regarding enzymatic activity, SOD and CAT increased in the dry season compared to the rainy season in 2012 and 2013. A higher increase in SOD was observed in 2013 and in CAT in 2012. APX, unlike the other enzymes, decreased by 72% in the dry season compared to the rainy season in 2013 (Figures 6E,G,I).

In the seacoast region, MDA content was only higher in the dry season in 2013. H_2_O_2_ had inverse patterns between the years, increasing by 16% in 2012 and decreasing by 15% in 2013 in the dry season compared to the rainy season (Figures 6B,D). Regarding enzymatic activity, SOD and CAT differed only in 2013; SOD decreased by 36% in the dry season in relation to the rainy season, while CAT increased by 72% in relation to the rainy season. APX differed in the different seasons in both years, decreasing by 93% in 2012 and increasing by 3.6 times in 2013 in the dry season in relation to the rainy season (Figures 6F,H,J).

In the semi-arid zone, only P content increased in the dry season (27%) compared to the rainy season, while N, K, and Na decreased (19, 36, and 28%, respectively) in 2012. In 2013, N, Na, and Cl had lower contents under dry-season conditions in relation to the rainy-season contents, dropping by around 25, 36, and 12%, respectively (Supplementary Figures S2A,C,E,G,I).

In the seacoast region, N and Cl content decreased by 12% (2012) and 33% (2013), respectively, in the dry season. Increases were observed in the dry season for P (34% in 2012 and 21% in 2013), K (18% in 2012), and Cl (28% in 2012) in relation to the rainy season (Supplementary Figures S2B,D,F,H,J).

### Leaf Anatomy

The dry season caused changes in the anatomical attributes of leaves. In the semiarid region, LT, MT, PPT, and SPT increased in the dry season (*P* < 0.05) in 2012 and decreased (*P* < 0.05) in 2013 compared to the rainy season (Table 3). On the other hand, LT, MT, and PPT decreased (*P* < 0.05) in the dry season of both years at the seacoast compared to the rainy season; SPT decreased only in 2013 (Table 3).

**TABLE 3:** The leaf anatomy traits of leaf thickness (LT), mesophyll thickness (MT), palisade parenchyma thickness (PPT), and spongy parenchyma thickness (SPT) in *Calotropis procera* during drought and rainy seasons (2012 and 2013) in semi-arid and seacoast regions (*n* = 4 ± SE).

**Region**	**Season**	**Year**	**LT (μm)**	**MT (μm)**	**PPT (μm)**	**SPT (μm)**
Semi-arid	Drought	2012	487 ± 2.7 C	459 ± 2.5 C	142 ± 1.9 C	317 ± 2.7 C
		2013	613 ± 0.9 B	550 ± 0.8 B	191 ± 3.5 B	360 ± 3.9 B
	Rainy	2012	391 ± 1.5 D	351 ± 0.6 D	106 ± 0.4 D	245 ± 1.0 D
		2013	695 ± 8.9 A	619 ± 8.5 A	218 ± 2.8 A	405 ± 7.6 A
Seacoast	Drought	2012	457 ± 1.7 D	397 ± 7.9 D	112 ± 0.4 D	285 ± 7.7 D
		2013	734 ± 20.6 B	677 ± 18.4 B	213 ± 11.4 B	463 ± 7.1 B
	Rainy	2012	486 ± 0.5 C	441 ± 0.5 C	141 ± 0.3 C	300 ± 0.4 C
		2013	844 ± 13.6 A	777 ± 15.5 A	273 ± 4.0 A	503 ± 17.6 A

*Means followed by different letters are significantly different according to the Student-Newman-Keuls test (*P* < 0.05).*

In the semi-arid region, the CT of abaxial and the ET of adaxial surfaces decreased during the dry season in both years. However, ECD decreased in both surfaces. In relation to stomatal measurements, SD had inverse patterns during the dry seasons of 2012 and 2013. This parameter decreased in adaxial surfaces in 2012 and increased in 2013 compared to the rainy season, while SI increased for abaxial and adaxial surfaces in 2012 in the dry season. For measurements of trichomes, TD decreased in abaxial surfaces in 2012 and adaxial surfaces in 2013 and increased in abaxial surfaces in 2013 in the dry season compared to the rainy season, while TI increased for abaxial and adaxial surfaces in 2013 and 2012, respectively, in the dry season compared to the rainy season (Supplementary Table S1).

In the seacoast region, CT increased for abaxial and adaxial surfaces in the dry season of 2012 compared to the rainy season, while ET decreased for adaxial surfaces in 2013. The ECD increased for both surfaces in the dry season of 2013 compared to the rainy season. Regarding the stomatal parameters, SD increased on adaxial surfaces in the dry season of 2013 compared to the rainy season. On the other hand, while SI increased on adaxial surfaces in 2012, it decreased on abaxial surfaces in 2013. In relation to trichome measurements, TD and TI increased for abaxial and adaxial surfaces in the dry season compared to the rainy season in both years (Supplementary Table S1).

### Principal Component Analysis

**FIGURE 7:**
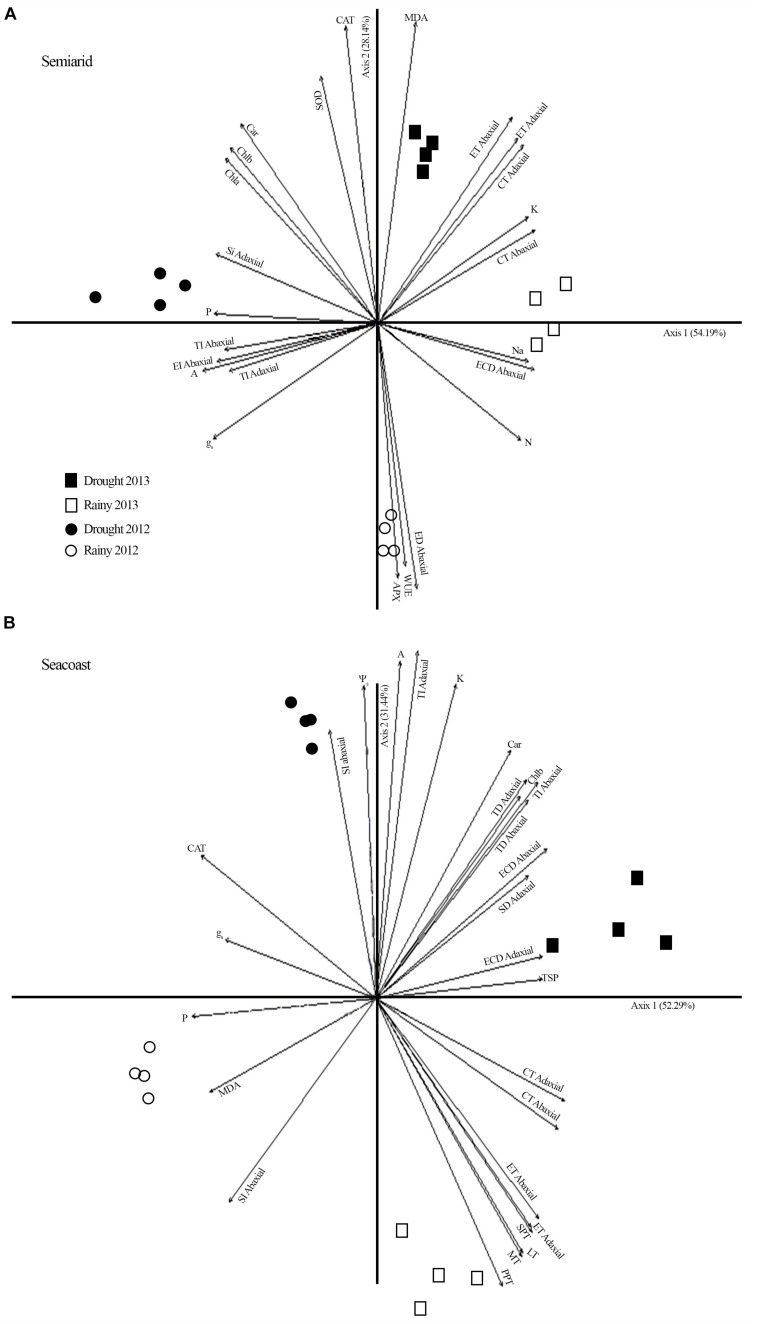
Principal component analysis (PCA) including potential osmotic (Table 2), gas exchange (Figure 3), biochemistry (Figures 4, 5), oxidative stress (Figure 6), and nutrients (Supplementary Figure S2) in *Calotropis procera* under semiarid and seacoast environment in rainy and drought seasons of 2012 and 2013. Vectors with values above 0.70 of correlation were represented.

PCA for the semi-arid region showed a water gradient composed of the 2012 drought (1.6 mm), 2013 drought (3.9 mm), and 2013 rainy season (38 mm) (Figure 7A). This indicates that less rainfall led to more fructose, reducing sugars, photosynthetic pigment accumulation, CAT, and SOD activity. This suggests that the antioxidant function of sugars and carotenoids favored protection of the photosynthetic apparatus to maintain gas exchange. In addition, the multivariate analysis pointed to a separation among the rainy season of 2012 and the other groups, exhibiting higher WUE, lower oxidative damage in the plasma membrane, and reduction in CAT and SOD activity.

At the seacoast, PCA multivariate analysis indicated a water gradient composed of the 2012 drought (5 mm), 2013 drought (58.3 mm), 2013 rainy season (274 mm), and 2012 rainy season (381.5 mm) (Figure 7B). This indicates that less rain led to more gas exchange at 1330 h and greater adjustment in the leaf anatomy, especially TD, which would aid in the dissipation of energy and minimize the water loss.

## Discussion

### General

Regardless of the season or the study site, our results showed that the daily curve of gas exchange in *C. procera* had similar behavior under dry season conditions, with the highest photosynthetic rates at both sites (Figure 3). *Calotropis procera* had decreased CO_2_ assimilation during the day only in the rainy season in a semi-arid region, while lower *g*_s_ values were measured, suggesting stomatal limitation (Tezara et al., 2011). In general, evergreen C_3_ metabolism plants in semi-arid and arid environments are uncommon (Lawson et al., 2014; Niinemets and Keenan, 2014; Kropp and Ogle, 2015). This photosynthetic performance in *C. procera*, even under prolonged drought, may have been supported by high CO_2_ mesophilic conductance, such as in shown in plants of the same species submitted to water deficit under controlled conditions (Rivas et al., 2017). We believe that changes in leaf anatomy under dry-season conditions at both the studied sites would support this hypothesis of high CO_2_ diffusion in the intercellular space (Table 3), except in the driest year (2012). Thus, leaves are thicker compared to those of equivalent age in the dry season. This would be one of the traits that maintain photosynthetic performance even under water restriction in the soil.

Negative soil water balance during dry seasons is an unfavorable scenario for most C_3_ plants (Santos et al., 2014; Figueiredo-Lima et al., 2018a; Figueiredo-lima et al., 2018b). However, *C. procera* showed tolerance and maintained high gas exchange rates by altering its biochemical metabolism and anatomical structures in both environments with high plasticity. These findings are an advance in the frontier of knowledge and reinforce the idea of multiple traits of different natures occurring in this species to support its tolerance to different environmental conditions.

The first question of this study was: do *C. procera* plants invest in the same morpho-anatomical traits in both environments? Not exactly; the morpho-anatomic traits measured revealed different changes in the semi-arid and littoral regions. In the semi-arid region, *C. procera* changed its leaf anatomy, which can maximize CO_2_ assimilation, with thicker mesophyll, high stomatal density, and greater efficiency of mesophilic conductance (Rivas et al., 2017), while *g*_s_ decreased in this place. For instance, this increase in mesophyll thickness is important for organizing chloroplasts on the cell surface by optimizing the CO_2_ uptake process (Oguchi et al., 2003). At the seacoast, even under high annual precipitation, leaves had increased cuticle thickness and trichome density in the dry season. These two traits are related to the incident light reflection process (Galmés et al., 2007) and reduced water loss to the atmosphere, as the cuticle acts as a hydrophobic barrier (Kerstiens, 1996), and trichomes promote permanence in the border layer (Burkhardt et al., 2012). These changes provide a favorable leaf microclimate and protection of the photosynthetic machinery. Our results accord with the higher density of trichomes found in *C. procera* in a South American site (Tezara et al., 2011). Another previous study that compared two species of *Encelia*, both with C_3_ photosynthetic metabolism, found no difference in photosynthetic rate. Although the native environment is different, one of them is more arid and hotter. The authors pointed out as one of the causes to CO_2_ assimilation maintenance that there is a high incidence of hair on the leaves of native species of the most arid and warm environment (Ehleringer and Bjorkman, 1978).

The second question was: does the leaf biochemistry differently change over the seasons in the two environments, and does this change lead to increased CO_2_ assimilation? Our results showed that these changes were different in the two studied places, and also, the measured traits point to a support of CO_2_ assimilation. The evergreen *C. procera* showed hardening performance under prolonged drought in the semi-arid region, since the soil water balance was always negative during the 24 months of the study (Walter et al., 2011; Rivas et al., 2013). High CO_2_ assimilation is supported by several traits, such as the abundance of photosynthetic pigments. In this study, both the chlorophyll and carotenoid contents were higher in the semi-arid region during the dry seasons. The higher levels of chlorophyll under low water availability would enable a high photosynthetic rate to be maintained, as well as acting as an electron drain (Lawlor and Cornic, 2002). Indeed, carotenoids are the major plant photoprotective molecules, acting in the dissipation of excess energy, such as the xanthophyll cycle (Domonkos et al., 2013; Esteban et al., 2015), acting as a singlet oxygen sink (Cazzaniga et al., 2012), and finally, promoting memory for light stress in the short and long term (García-Plazaola et al., 2012; Esteban et al., 2015). Therefore, these chloroplastidic pigments are linked to leaf nitrogen nutrition, which decreases in most plant species under conditions of soil water deficiency. In fact, mineral nutrition is another modulating factor for photosynthetic performance (Frosi et al., 2013). This increase in chlorophyll concentrations has been related to uptake and maintenance of leaf P and N content (Ruiz-Sánchez et al., 2010). In the present work, leaves retained nitrogen, phosphorus, and potassium during both dry seasons, showing contents very close to those in the rainy season, which may have contributed to the high photosynthetic rates. In addition, phosphorus is a constituent of NADPH and ATP, which are necessary for CO_2_ uptake and regeneration of ribulose 1,5-biphosphate (RuBP), leading to high photosynthetic rates (Warren, 2011; Singh et al., 2013).

Photosynthetic pigments, nutrients, and soluble sugars play an important role in environmental stress tolerance by supporting photosynthetic activity. In *C. procera*, high leaf content of sucrose, fructose, and reducing sugars in the dry season under a semi-arid climate may act as signaling, an electron sink, and a ROS neutralizer, thus avoiding oxidative stress under the low water availability, >35°C air temperature (Supplementary Figures S3, S4), and excess light of a semi-arid environment (Ramel et al., 2009; Filek et al., 2015). Furthermore, non-structural carbohydrates produced in the photosynthetic process are supports to metabolism and growth, for example, sugars used for respiration (Carbone and Trumbore, 2007).

Despite all these traits, in the driest year in the semi-arid region (2012), plants showed an increase in the leaf content of MDA, which is generated from lipid peroxidation of the cell membranes through ROS. Simultaneously, two antioxidant system enzymes, SOD and CAT, showed higher activity compared with the rainy season in the same place. The first is considered to be the strongest antioxidant enzyme involved in the tolerance process, being the main line of defense against ROS (Sayfzadeh et al., 2011). These enzymes act on superoxide radicals and subsequently decrease the risk of hydroxyl radical formation (Gill and Tuteja, 2010). H_2_O_2_ is a less reactive species and can be metabolized by CAT. Indeed, higher CAT activity has been observed in dry seasons, which could also be related to increased photorespiration, which has H_2_O_2_ in the peroxisome as an intermediate product (Hagemann et al., 2013). In fact, C_3_ evergreen desert plants have high photorespiration throughout the day, and this represents a strong sink to excess energy from sunlight and under an air temperature higher 35°C (Tezara et al., 2011; Lawson et al., 2014). Moreover, photosynthetic activity is recognized as one of the most effective sinks of excess light energy and means to avoid its consequences (Lawlor and Cornic, 2002). Thus, maintaining high CO_2_ assimilation even under limited soil water could be strategic for evergreen C_3_ plants (Tezara et al., 2011; Frosi et al., 2013).

The CO_2_ assimilation in seacoast plants was similar in both seasons, as in the semi-arid region. In this environment, VPD values were 50% lower than those found in the semi-arid region. Additionally, there was a positive soil water balance in the rainy season. Seacoast plants did not show an increase in photosynthetic pigments in dry seasons when the soil water balance was negative. Therefore, the leaf nitrogen and phosphorus content remained stable. The antioxidative system was modulated even under greater precipitation and lower VPD, in that SOD, CAT, and APX changed their activities between the dry and rainy seasons in this environment along the coast. Contrary to the stability of the mineral nutrient content in the leaves found in the semi-arid environment, some foliar mineral nutrients showed major reductions under seacoast conditions, such as decreased leaf potassium content and three times higher Na^+^ values throughout the study period compared with the values of semi-arid plants. The Na^+^/K^+^ ratio in seacoast plants was higher than in semi-arid plants despite this photosynthetic metabolism not having changed. In general, Na^+^ causes ionic imbalance and damages the chloroplast structure (Gomes et al., 2011). Our results suggest that *C. procera* would neutralize Na^+^ in leaves (Cantrell and Linderman, 2001) before it arrived at chloroplasts.

The third question was: does the photosynthetic activity have the same intensity in both rainy and dry seasons? Partially, since only the CO_2_ assimilation curves for the semi-arid environment were similar during the two dry seasons studied, while stomatal conductance was different at those times. On the other hand, in the seacoast region, both variables, *g*_s_ and *A* were similar, while the highest values were observed in the dry season, as was found in the semi-arid region. Finally, *C. procera* showed a balance among its traits at the coast, with the antioxidative system, sugar metabolism, and leaf morpho-anatomy related to light reflection and maintenance of the microclimate favoring photosynthetic machinery protection.

The fourth question was: what are the traits that determine the photosynthetic metabolism performance? *C. procera* showed several changes to tolerate the environmental conditions in both studied sites. Indeed, some adjustment is common in both environments, although the water regime is completely different between semi-arid and seacoast regions. Thus, ecophysiological changes in leaf traits from both studied environments, such as osmotic potential, sugar metabolism, and antioxidative system, should lead to high photosynthetic capacity. Moreover, several morpho-anatomical changes, including in leaf thickness, stomatal index and density, and index of trichomes in leaves developed under rainy or dry season were exhibited. These changes allowed photosynthesis to remain high in the dry period under a semi-arid environment, even at the maximum point of VPD and PPFD and with an air temperature above 35°C.

## Conclusion

In conclusion, the present work had as its main goal the investigation of what physiological and anatomical attributes allow *C. procera* to maintain high performance of photosynthetic metabolism activity even under high VPD and low soil water availability. This study is an advance in the understanding of C_3_ performance under limited soil water. *Calotropis procera* invested in different leaf morpho-anatomical traits during the rainy and dry seasons, as well as between the two places studied, such as having greater stomatal and trichome densities in the adaxial epidermis, which would have given support to the always low stomatal conductance. Additionally, these traits possibly maintained the stability of the leaf water status, as indicated by the low variation in foliar osmotic potential, even under strong differences in soil water balance between locations and seasons. Our results suggest that these traits could support the maintenance of high photosynthetic performance, since the CO_2_ assimilation curves under a semi-arid environment were similar during both dry seasons studied, although stomatal conductance was different than CO_2_ assimilation at the same time. On the other hand, in the seacoast region, both variables, *g*_s_ and *A* were similar, while the highest values were observed under dry season, as was found for the semi-arid region. Furthermore, an efficient antioxidative system and leaf sugar and photosynthetic pigment content dynamics would contribute to photosynthetic machinery protection, even in a dry season and under high light incidence. Our study differs from previous ones on the same species because measurements were taken throughout the day, and also leaves were collected at the same time to investigate different variables under field conditions, which allowed the observation of a broad scenario of photosynthetic performance for *C. procera*, a C_3_ evergreen species.

## Data Availability Statement

The datasets generated for this study are available on request to the corresponding author.

## Author Contributions

RR and MS designed the project. RR and HF conducted the gas exchange and sample collection. RR and VB conducted the biochemical analysis. RR and EA performed the leaf anatomical measurements. RR, GF, and MS wrote the manuscript. All authors contributed to the article and approved the submitted version.

## Conflict of Interest

The authors declare that the research was conducted in the absence of any commercial or financial relationships that could be construed as a potential conflict of interest.

## Supplementary Material

FIGURE S1 **(A,B)** Free amino acids (FAA), **(C,D)** proline, and **(E,F)** total soluble protein (TSP) in *Calotropis procera* under semi-arid and seacoast environments in rainy and drought seasons of 2012 and 2013 (*n* = 4 ± SE). Values followed by different letters differ from each other according to the Student-Newman-Keuls test (*P* < 0.05).

FIGURE S2 **(A,B)** Nitrogen (N), **(C,D)** phosphorus (P), **(E,F)** potassium (K), **(G,H)** sodium (Na), and chlorine (Cl) in *Calotropis procera* under semi-arid and seacoast environments in rainy and drought seasons of 2012 and 2013 (*n* = 4 ± SE). Values followed by different letters differ from each other according to the Student-Newman-Keuls test (*P* < 0.05).

FIGURE S3 Diurnal temperature changes on the days of gas exchange measurement at the semi-arid **(A)** and seacoast **(B)** locations studied.

FIGURE S4 Average monthly temperature during the years 2012 and 2013 at the semi-arid **(A)** and seacoast **(B)** locations. Average daily temperature on the dates of data collection in the semi-arid **(C)** and seacoast **(D)** regions.
