# Corrigendum: Ecophysiological Traits of Invasive C_3_ Species *Calotropis procera* to Maintain High Photosynthetic Performance Under High VPD and Low Soil Water Balance in Semi-Arid and Seacoast Zones

**DOI:** 10.3389/fpls.2020.611685

**Published:** 2020-11-11

**Authors:** Rebeca Rivas, Vanessa Barros, Hiram Falcão, Gabriella Frosi, Emília Arruda, Mauro Santos

**Affiliations:** ^1^Laboratório de Fisiologia Vegetal, Departamento de Botânica, Universidade Federal de Pernambuco, Recife, Brazil; ^2^Laboratório de Anatomia Vegetal, Departamento de Botânica, Universidade Federal de Pernambuco, Recife, Brazil

**Keywords:** C_3_ photosynthesis, evergreen, leaf anatomy, oxidative stress, plant biomass, sugars metabolism

In the original article, there was a mistake in the legend for Table 2 as published. **Osmotic potential as demonstrated in the equation in the material and methods is the negative value**. The correct legend appears below.

1. The fractionation of the sugars was removed due to duplicate results, probably caused by contamination of the samples. However, the presentation of soluble sugars and starch allows to maintain the same discussion together with the other results.
  In the original article, there was a mistake in the legend for Figure 4 as published. The correct legend appears below.
2. With the removal of the fractionation of sugars, Figure 7 was changed, as the PCA is a synthesis of the analyzed variables.

**Table 2:** Osmotic potential (−Ψ_S_) in *Calotropis procera* during drought and rainy seasons (2012 and 2013) in semi-arid and seacoast regions (*n* = 4 ± SE).

**Location**	**Season**	**Year**	**–Ψ_S_ (MPa)**
Semiarid	Drought	2012	1.33 ± 0.03 AB
		2013	1.41 ± 0.02 B
	Rainy	2012	1.34 ± 0.04 AB
		2013	1.22 ± 0.03 A
Seacoast	Drought	2012	1.07 ± 0.03 NS
		2013	1.14 ± 0.03
	Rainy	2012	1.18 ± 0.02
		2013	1.20 ± 0.04

*Means followed by different letters are significantly different according to the Student-Newman-Keuls test (P < 0.05)*.

**Figure 4:**
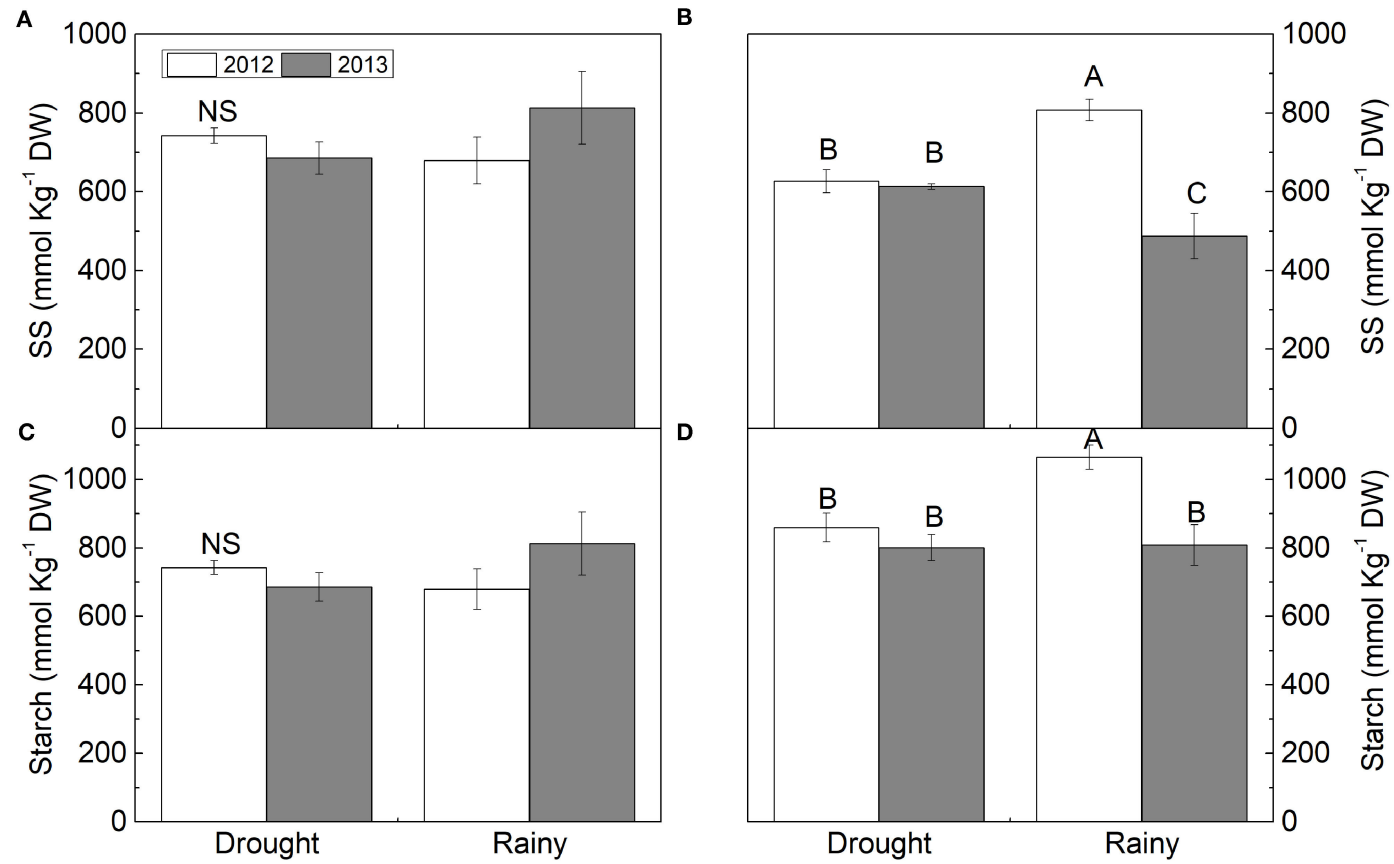
**(A,B)** soluble sugar (SS) and **(C,D)** starch in *Calotropis procera* under semiarid and seacoast environment in rainy and drought seasons of 2012 and 2013 (*n* = 4 ± S.E.). Values followed by different letters differ from each other by the Student Newman Keuls test (*P* < 0.05).

**Figure 7:**
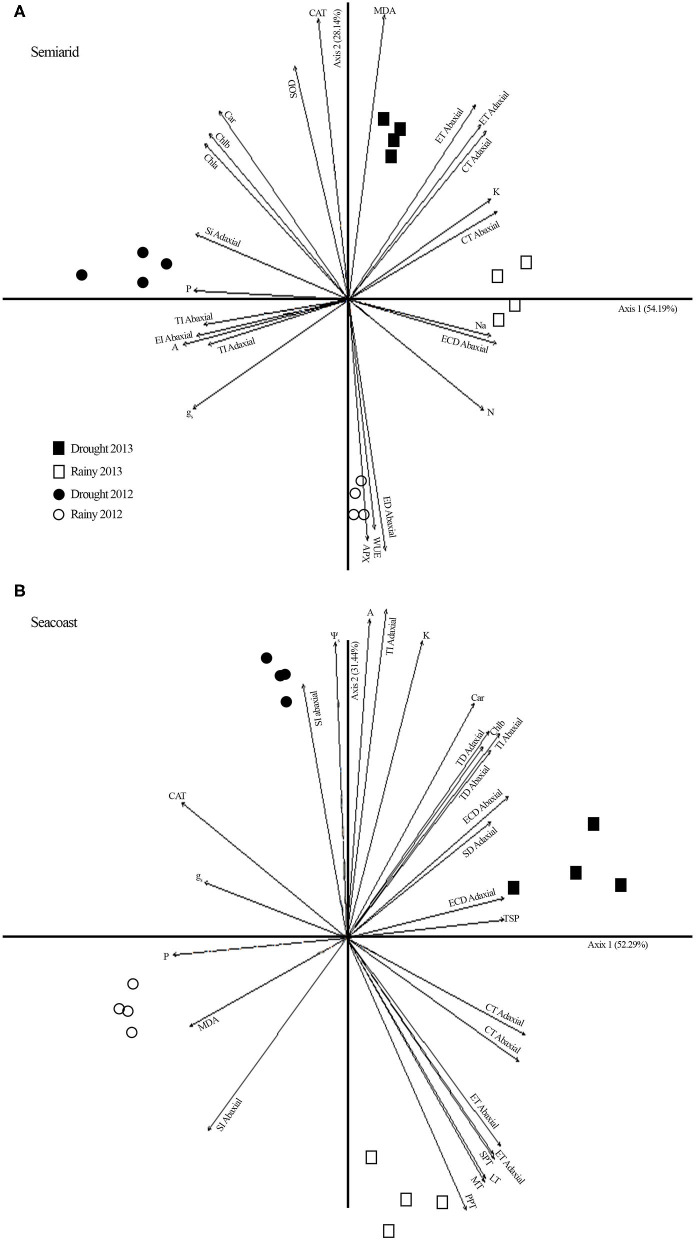
Principal component analysis (PCA) including potential osmotic (Table 2), gas exchange (**Figure 3**), biochemistry (Figures 4, **5**), oxidative stress (**Figure 6**), and nutrients (**Figure S2**) in *Calotropis procera* under semiarid and seacoast environment in rainy and drought seasons of 2012 and 2013. Vectors with values above 0.70 of correlation were represented.

In the original article, there was a mistake in the legend for Figure 7 as published. The correct legend appears below.

The authors apologize for this error and state that this does not change the scientific conclusions of the article in any way. The original article has been updated.

