# Supplementary material for: Ecophysiological Traits of Invasive C3 Species Calotropis procera to Maintain High Photosynthetic Performance Under High VPD and Low Soil Water Balance in Semi-Arid and Seacoast Zones
Source: Front Plant Sci. 2020 Jul 2;11:717. doi: 10.3389/fpls.2020.00717 (PMC7343903; doi:10.3389/fpls.2020.00717)
Supplement: Supplementary file 5 [file Table_1.DOCX]

**Table S1.** Cuticle and epidermis traits Cuticle thickness (CT), Epidermis thickness (ET), Epidermal cell density (ECD), Stomatal density (SD), Stomatal index (SI), Trichome Density (TD), Trichome index (TI) in *Calotropis procera* during drought and rainy seasons (2012 and 2013) in semiarid and seacoast regions (n = 4 ± s.e.). Means followed by different letters are signiﬁcantly different by Student Newman Keuls (P < 0.05).

| Region | Season | Year | Surface | CT (µm) | ET (µm) | ECD (0.02mm^2^) | SD (0.02mm^2^) | SI (%) | TD (0.02mm^2^) | TI (%) |
| --- | --- | --- | --- | --- | --- | --- | --- | --- | --- | --- |
| Semiarid | Drought | 2012 | Abaxial | 3.06 ± 0.03 D | 12.38 ± 0.07 D | 120.85 ± 1.49 E | 10.40 ± 0.21 E | 9.95 ± 0.74 B | 11.41 ± 0.30 D | 11.16 ± 0.86 A |
|  |  | 2013 | Abaxial | 4.45 ± 0.03 B | 20.58 ± 0.42 C | 157.37 ± 0.72 AB | 10.67 ± 0.14 E | 6.40 ± 0.09 D | 16.67 ± 0.14 B | 9.54 ± 0.06 A |
|  | Rainy | 2012 | Abaxial | 3.63 ± 0.01 C | 11.91 ± 0.09 D | 157.80 ± 0.39 AB | 12.51 ± 0.03 AB | 7.40 ± 0.3 C | 17.85 ± 0.42 A | 9.98 ± 0.21 A |
|  |  | 2013 | Abaxial | 5.37 ± 0.40 A | 21.53 ± 0.76 C | 153.98 ± 3.71 ABC | 10.87 ± 0.16 DE | 6.62 ± 0.3 CD | 12.95 ± 0.44 C | 7.76 ± 0.23 B |
|  | Drought | 2012 | Adaxial | 3.14 ± 0.04 D | 13.34 ± 0.18 D | 135.20 ± 3.67 D | 12.73 ± 0.16 A | 11.06 ± 0.79 A | 12.45 ± 0.29 CD | 11.02 ± 0.80 A |
|  |  | 2013 | Adaxial | 4.9 ± 0.13 A | 24.74 ± 1.3 B | 148.80 ± 1.39 CB | 12.40 ± 0.17 AB | 7.71 ± 0.13 CD | 10.17 ± 0.15 E | 6.37 ± 0.11 B |
|  | Rainy | 2012 | Adaxial | 3.26 ± 0.04 D | 13.9 ± 0.05 D | 147.13 ± 4.21 C | 11.38 ± 0.19 D | 7.13 ± 0.08 CD | 12.60 ± 0.44 CD | 7.79 ± 0.17 B |
|  |  | 2013 | Adaxial | 5.34 ± 0.19 A | 26.83 ± 0.65 A | 161.50 ± 9.67 A | 12.00 ± 0.27 B | 7.01 ± 0.07 CD | 12.55 ± 0.49 CD | 7.48 ± 0.37 B |
| Seacoast | Drought | 2012 | Abaxial | 3.38 ± 0.02 D | 10.35 ± 0.10 E | 123.10 ± 0.23 C | 11.17 ± 0.31 B | 8.32 ± 0.19 BC | 13.72 ± 0.33 B | 9.91 ± 0.15 A |
|  |  | 2013 | Abaxial | 5.15 ± 0.07 B | 19.77 ± 0.60 C | 139.65 ± 2.34 B | 11.33 ± 0.05 B | 7.60 ± 0.14 C | 16.00 ± 0.56 A | 10.23 ± 0.16 A |
|  | Rainy | 2012 | Abaxial | 2.88 ± 0.02 E | 10.44 ± 0.13 E | 115.18 ± 2.03 C | 11.27 ± 0.02 B | 9.03 ± 0.09 B | 8.75 ± 0.25 D | 6.57 ± 0.12 D |
|  |  | 2013 | Abaxial | 5.00 ± 0.32 B | 21.56 ± 0.27 C | 118.87 ± 1.44 C | 11.17 ± 0.21 B | 8.88 ± 0.11 B | 11.13 ± 0.56 C | 8.70 ± 0.43 B |
|  | Drought | 2012 | Adaxial | 3.93 ± 0.01 C | 13.75 ± 0.23 D | 115.22 ± 2.06 C | 11.28 ± 0.22 B | 10.54 ± 0.67 A | 8.68 ± 0.63 D | 8.10 ± 0.12 C |
|  |  | 2013 | Adaxial | 5.87 ± 0.10 A | 24.81 ± 1.94 B | 168.63 ± 19.54 A | 14.53 ± 1.21 A | 8.04 ± 0.25 BC | 11.57 ± 0.38 C | 6.50 ± 0.17 D |
|  | Rainy | 2012 | Adaxial | 2.97 ± 0.04 E | 14.90 ± 0.04 D | 116.10 ± 0.50 C | 10.33 ± 0.10 B | 8.19 ± 0.05 BC | 5.70 ± 0.05 E | 4.66 ± 0.03 E |
|  |  | 2013 | Adaxial | 5.69 ± 0.09 A | 27.03 ± 0.26 A | 122.43 ± 0.81 C | 10.63 ± 0.10 B | 8.04 ± 0.08 BC | 6.17 ± 0.15 E | 4.81 ± 0.11 E |

**Table S2.** Spearman’s correlation between *A* and g_s_; generalized linear model (GLM) between *A* and DPV, *A* and PPFD, g_s_ and DPV, g_s_ and PPFD. In *Calotropis procera* during drought and rainy seasons (2012 and 2013) in semiarid and seacoast regions (n = 4). Values in bold means P < 0.05.

| Region | Season | Year | *A* x g_s_ | | *A* x DPV | | *A* x PPFD | | g_s_ x DPV | | g_s_ x PPFD | |
| --- | --- | --- | --- | --- | --- | --- | --- | --- | --- | --- | --- | --- |
|  |  |  | R | P | R | P | R | P | R | P | R | P |
| Semiarid | Drought | 2012 | 0.88 | 0.00 | 0.31 | 0.00 | 0.62 | 0.00 | 0.2 | 0.00 | 0.79 | 0.00 |
|  |  | 2013 | 0.93 | 0.00 | 0.02 | 0.12 | 0.67 | 0.00 | -0.02 | 0.7 | 0.45 | 0.00 |
|  | Rainy | 2012 | 0.8 | 0.00 | 0.52 | 0.00 | 0.83 | 0.00 | 0.01 | 0.24 | 0.11 | 0.00 |
|  |  | 2013 | 0.73 | 0.00 | 0.35 | 0.00 | 0.77 | 0.00 | 0.03 | 0.06 | 0.37 | 0.00 |
| Seacoast | Drought | 2012 | 0.83 | 0.00 | 0.75 | 0.00 | 0.87 | 0.00 | 0.42 | 0.00 | 0.82 | 0.00 |
|  |  | 2013 | 0.76 | 0.00 | 0.69 | 0.00 | 0.95 | 0.00 | 0.54 | 0.00 | 0.87 | 0.00 |
|  | Rainy | 2012 | 0.91 | 0.00 | 0.34 | 0.00 | 0.76 | 0.00 | 0.31 | 0.00 | 0.81 | 0.00 |
|  |  | 2013 | 0.92 | 0.00 | 0.37 | 0.00 | 0.87 | 0.00 | 0.43 | 0.00 | 0.9 | 0.00 |
